# Fucoidans: a new frontier in brown seaweed research and biotechnology

**DOI:** 10.1016/j.tcsw.2026.100179

**Published:** 2026-07-09

**Authors:** Thierry Tonon, Finn L. Aachmann, Vincent Ferrières, Cécile Hervé

**Affiliations:** a Centre for Novel Agricultural Product, Department of Biology, University of York, Wentworth Way, York YO10 5DD, United Kingdom; b Department of Biotechnology and Food Science, Norwegian University of Science and Technology (NTNU), NO-7491 Trondheim, Norway; c Univ Rennes, Ecole Nationale Supérieure de Chimie de Rennes, CNRS, ISCR – UMR 6226, F-35000 Rennes, France; d CNRS/Sorbonne Université, Station Biologique De Roscoff, Place Georges Teissier, 29680 Roscoff, France

**Keywords:** Fucoidans, Brown algae, Sulfated polysaccharides and oligosaccharides, Seaweed biotechnology, Bioactive compounds

## Abstract

Fucoidans, also known as fucose-containing sulfated polysaccharides (FCSPs), are abundant and structurally complex glycans found in the cell wall of brown algae. These compounds have been shown to have fundamental roles in the development and physiology of these organisms, to provide a major contribution to marine carbon sequestration, and to exhibit numerous bioactivities. Despite this, we currently have a limited understanding of the metabolic pathways and enzymes involved in synthesis and remodelling of fucoidans, their spatial and temporal dynamics in brown algae and in the marine environment, and how their structure and composition influence their bioactivities. Here, we highlight some of the challenges that have hindered progress on fucoidan research. We then moved on to underline some of the recent technical progress and new resources that would contribute to alleviate these challenges when combined together. This includes new technical approaches in analytical chemistry and glycobiology, as well the extension of genomic resources and genetics tools for brown algae. Finally, we suggest how progress in fucoidan research should contribute to advance understanding on brown algae biology, while supporting current and developing new aspects in fucoidan applications and seaweed biotechnology as part of the growing blue bioeconomy.

## 1 Why sulfated polysaccharides of brown algae need to be investigated further?

Seaweed, or macroalgae, are a rich and yet underutilised biomass, with strong potential to address current global challenges, including climate change, global food demand, and rising need for natural bioactive compounds. Seaweed cultivation is expanding, with production representing over 38 million tons per year (FAO, 2024). In addition, seaweed blooms and invasions increase globally, as illustrated by the recurrent sargassum blooms impacting the Caribbean and West Africa since 2011 (Marsh et al., 2023). Despite the growing importance of seaweed in our society, we currently have a scarce understanding of their biology, including their basic primary metabolism, and we urgently need to address this knowledge gap.

**Fig. 1:**
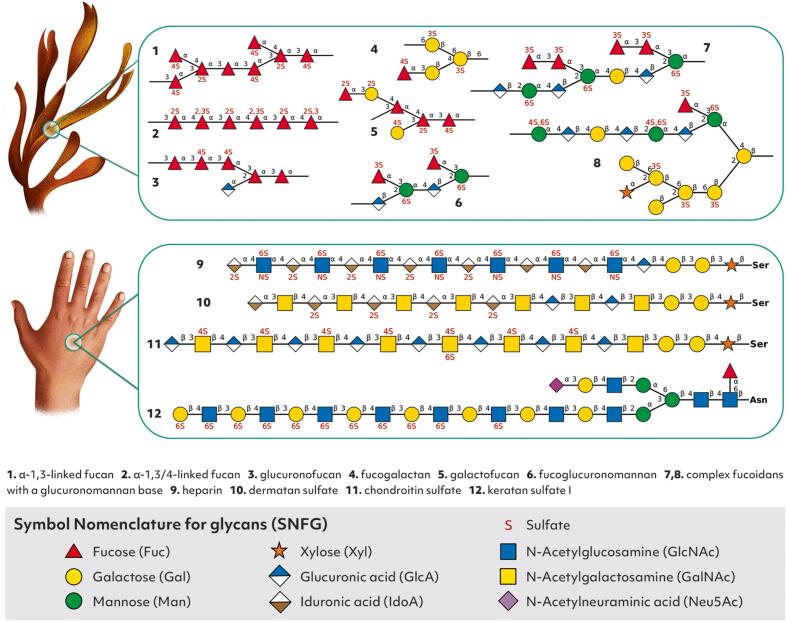
Selected chemical structures of brown algal FCSPs and human GAGs. (For interpretation of the references to colour in this figure legend, the reader is referred to the web version of this article.)

Macroalgae inhabit highly dynamic ecosystems, and several molecular evolutionary events have shaped their metabolic network. One critical example is the biosynthesis and re-modelling of the cell-surface glycans found within the seaweed extracellular matrices (ECMs, or cell-walls). These polysaccharides are major structural, functional, and industrially exploited compounds, and a hotspot for innovation. Among them, the fucose containing sulfated polysaccharides (FCSPs or fucoidans) produced by brown algae have been notoriously difficult to study so far. Fucoidans account for approximately 9–14% of the total dry weight of these organisms (Nikolić-Chenais et al., 2022; Birgersson et al., 2024). These polysaccharides represent a complex mixture of anionic macromolecules including homopolymers of fucans with a backbone structure of sulfated fucose residues, and heterogeneous polymers harbouring non-fucose backbones (including sulfated and non-sulfated galactans, and glucuronomanans) with side branches of sulfated fucose (Fig. 1) (Deniaud-Bouët et al., 2017). Fucoidans mediate numerous physiological functions in brown algae such as cell desiccation resistance (Moebus et al., 1974), cell adhesion (Brawley and Quatrano, 1979), cell elongation (Bisgrove and Kropf, 2001; Siméon et al., 2020), cell differentiation and cell fate decisions (Kropf et al., 1993; Berger et al., 1994). Variations in FCSPs composition have been shown to be influenced by factors such as species, geographic location and abiotic factors (Ponce and Stortz, 2020). The bioactivities of fucoidans, including antiviral (Schaeffer and Krylov, 2000; Ponce et al., 2003; Ahmadi et al., 2015), anticancer (Atashrazm et al., 2015; Wu et al., 2016; Teas and Irhimeh, 2017), regulation of immune responses (Kopplin et al., 2018; Birgersson et al., 2024), and haemostasis properties (Ustyuzhanina et al., 2014; Mourão, 2015; Zaporozhets and Besednova, 2016) have been known and discussed for decades (Deniaud-Bouët et al., 2017). In line with this, the fucoidan market size is expected to grow significantly in the coming years based on current market growth reports. More recently, fucoidan secretion by brown algae has gained attention as a potential mechanism for carbon dioxide removal (Buck-Wiese et al., 2023). These polysaccharides are supposed to be long-lasting in marine sediments and recalcitrant to degradation as this process requires complex catabolic pathways featured only in highly specialised organisms (Sichert et al., 2020; Pérez-Cruz et al., 2024) and is influenced by phosphate availability (Xu et al., 2026).

The structure and biological roles of fucoidans in brown algae are reminiscent of the sulfated glycosaminoglycans (GAGs) in mammals. GAGs are a class of long linear sulfated polysaccharides covalently attached to multiple core proteins (Fig. 1). They include structurally and functionally diverse glycan chains such as chondroitin sulfate, heparan sulfate and keratan sulfate, consisting of N-sulfonylglucosamine, *N*-acetylglucosamine, and *N*-acetylgalactosamine residues alternating in glycosidic linkages with glucuronic acid, iduronic acid, or galactose residues (Rudd et al., 2010). These proteoglycans are surrounding all mammalian cell surfaces and are the major driving force for a large variety of essential cellular functions as mentioned above for the fucoidans. Their multifunctional properties are essentially mediated by their respective GAG moieties that includes diverse monosaccharide composition (see above) and sulfation patterns. The structural divergence of the GAG chains is enzymatically generated and strictly regulated by the corresponding biosynthetic machineries. Their sulfation patterns are cell type- and developmental stage-specific, serving as dynamic templates to promote or inhibit specific cellular interactions and signalling events. Disruption of the sulfate patterns can result in severe developmental abnormalities and growth of several cancer types (Wieboldt and Läubli, 2022). As mentioned above, the scientific literature is extensive on the bioactivities held by FCSPs and their derived oligosaccharides (FCSOs), with most of them stemming from their ability to mimic the carbohydrate moieties of GAGs.

Interestingly, the sulfation of polysaccharides such as fucoidans and GAGs is reminiscent of other well-known regulatory processes of biological importance such as protein phosphorylation and nucleic acid methylation. Phosphorylation is the most common post-translational modification of proteins and emerged as a universal and essential link in the chain of biochemical events that occurs in living systems (Cohen, 2002). Similarly, the first biological roles for DNA methylation were inferred from studies on bacteria, prior to the extension of comprehensive investigations in eukaryotic systems (Mattei et al., 2022). In contrast, considerably less is known about the naturally occurring sulfate modification of glycans in all organisms in general (Lima et al., 2022). In this context, advancing knowledge on fucoidans in brown algae will help getting a greater understanding of glycan sulfation in eukaryotes. It may even reveal parallels with phosphorylation and methylation, suggesting that sulfation represents a third, yet largely overlooked, mechanism of molecular regulation.

**Fig. 2:**
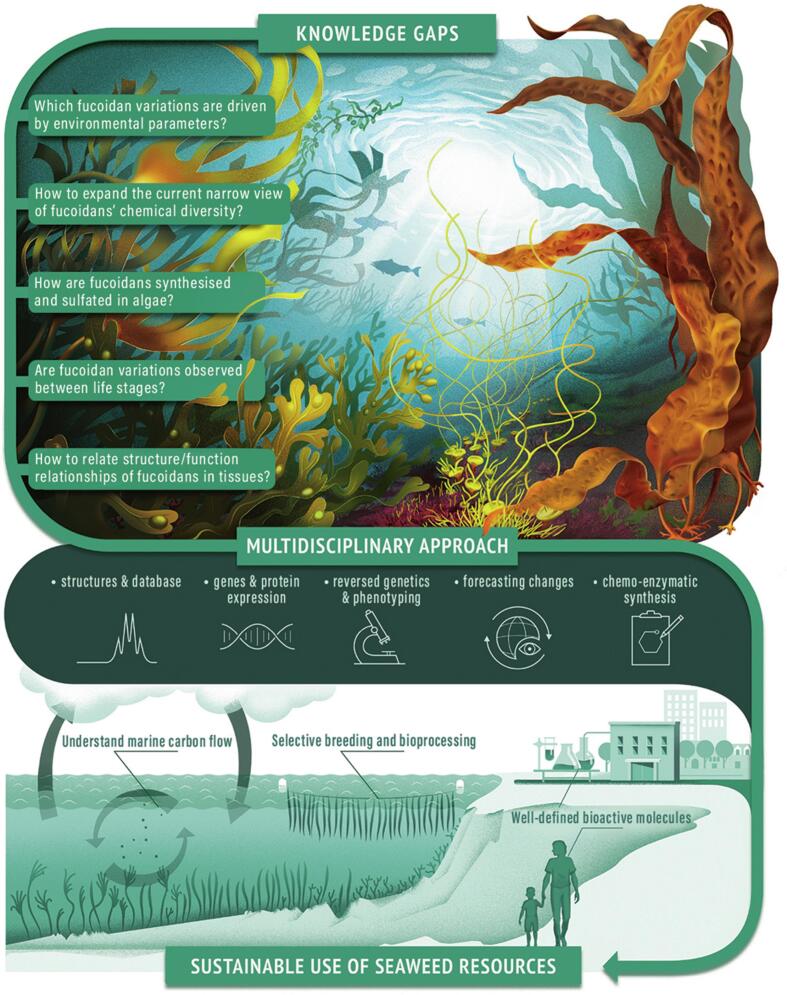
Identified knowledge gaps and description of a multidisciplinary approach to advance our understanding of FCSPs metabolism in brown algae for the benefit of Society. (For interpretation of the references to colour in this figure legend, the reader is referred to the web version of this article.)

Despite critical role of fucoidans in algal physiology and as bioactives, we currently have only limited understanding of how the content and structure of FCSPs change according to environmental conditions, how these variations impact their physiological roles and how they are made in brown algae (Fig. 2). Several factors can be brought forward to explain this lack of knowledge, including the limited number of species that have successfully been domesticated, as this hindered progress on cell wall research on algae cultivated in control conditions, but also the difficulty in extracting pure fucoidan molecules due to their polydisperse nature, and until very recently, the lack of genomic data and genetic techniques for brown algae. FCSPs have been studied only at the oligomer stage in a few brown algae, and robust structural information is still lacking. Moreover, little is known about the enzymatic biosynthesis and remodelling of FCSPs/FCSOs in brown algae. We consider it is time now to address these basic research knowledge gaps by considering growing interest in algal science, new resources and progress in technological approaches, and through synergistic and interdisciplinary research. Unveiling the molecular basis of fucoidan metabolism in brown algae, together with assessing structure-relationships of these compounds and their spatial-temporal variations in vivo is necessary, not only to extend our understanding of the rules of life, but also to foster seaweed biotechnology and support the blue bioeconomy (Fig. 2).

## 2 Towards in-depth characterization of fucoidan diversity and structure

Glycans are among the most complex biological molecules found in nature (Laine, 1994). They differ in their lengths and in their monosaccharide units that are organized in a variety of combinations and linkages. Glycans also feature diverse types of branching and functional groups and can be further modified by non-glycan entities to which they can be attached to. The complexity of many glycan structures requires an array of different methods for their analytical and structural analyses. FCSPs make no exception to the rule, their complexity and diversity being a challenge to their structural resolution. Over the past two decades, many different technologies have been developed for the extraction and analysis of glycans and glycoconjugates.

Most traditional methods for fucoidans extractions relied on incubating biomass under mild-acidic condition or in presence of hot water for several hours and at temperature ranging between 70 and 100 °C. However, the obtention of purified fractions of long and unmodified FCSPs is rarely achieved, essentially because extending the duration of the extraction process to improve its efficacy, leads to partial degradation and/or partial desulfation of the polymers (Flórez-Fernández et al., 2018; Birgersson et al., 2023). In this context, enzyme-assisted extraction protocols have been implemented recently, using enzymes such as cellulases, alginate lyases and proteases to degrade the cell wall of brown algae. It has been shown that both chemical and enzymatic methods produced fractions with different FCSPs content, composition, and degree of purity when tested in parallel on the same biomass (Nguyen et al., 2020; Machado et al., 2022; Birgersson et al., 2026). Following extraction, FCSPs can be fractionated by anionic exchange chromatography, and different fractions analysed by size-exclusion chromatography (SEC) coupled with multi-angle light scattering (MALS) to estimate their molecular weight and heterogeneity. In parallel, fractions are used for compositional and elemental analysis to determine the monosaccharide content and the level of sulfation of FCSPs. Gas chromatography-mass spectrometry (GC–MS) of partially methylated and acetylated alditols has also been deployed to determine the glycosidic linkages of non-sulfated glycans (Bajwa et al., 2024). However, these approaches provide only limited insights into the structure of FCSPs, and improving glycan annotation is key to study and understand structure-relationships of glycans.

High-resolution structural information of FCSPs, including the stereochemistry of carbohydrate hydroxyl groups and glycosidic linkages, as well as the degree and position of modifications and linkages, can be gained by Nuclear Magnetic Resonance (NMR) spectroscopy and Mass Spectrometry. NMR spectroscopy provides atomic-level resolution and has been used to elucidate the structural composition of glycans, as well as to some extent, their 3D structure. For glycan oligomers (6–10-mers), it is possible to achieve chemical shift resolution for the unique assignment of each atom in the oligomer. However, for longer oligosaccharides, factors such as monosaccharide composition, glycosidic linkages, branching, and chemical modifications (e.g. sulfation, acetylation, and glycosylation) can result in small chemical shift differences melting together. The slow molecular tumbling of FCSPs, meaning their reduced dynamic behaviour due to large molecular size, contributes to line broadening and a consequent loss of atomic resolution. In the case of FCSPs, reducing structural complexity through desulfation, deacetylation, and controlled degradation has been instrumental in establishing their backbone and side-chain structures in several brown algal species (Bilan et al., 2010 and 2017; Usoltseva et al., 2017; Bilan et al., 2018). Additionally, the removal of sulfate and acetyl groups allows for linkage analysis via methylation, enabling the determination of linkage positions within the sugar sequence and aiding in the characterization of the unmodified (“naked”) structure of FCSPs (Bilan et al., 2010 and 2008). More recently, repeating structural motifs in FCSPs have been identified and fully elucidated by NMR spectroscopy, providing insights into the presence of regular and recurring structural fragments within otherwise randomly sulfated and acetylated FCSP structures. (Tran et al., 2022; Trang et al., 2025; Reyes-Weiss et al., 2026). The development of carbohydrate-NMR databases such as GlyNest (Lütteke et al., 2006) and CASPER (Dorst and Widmalm, 2025), which contain experimental ^1^H and ^13^C NMR chemical shifts of diverse glycans, is instrumental to simulating and validate oligosaccharides structures. However, the predicted power is still limited to smaller oligomer fragments, although developments in using machine learning algorithms are appearing. Currently, no NMR database for fucoidan-containing sulfated oligomers/oligosaccharides (FCSOs) exists, despite published information on many examples of different 4–5-mer FCSOs (Bezerra et al., 2020; Shen et al., 2020; Tran et al., 2022; Liu et al., 2023; Shen et al., 2024; Chen et al., 2025; Trang et al., 2025). Establishing a database with chemically well-defined FCSOs using NMR data would enable the creation of a ^1^H,^13^C HSQC (heteronuclear single quantum coherence) fingerprint spectra library, facilitating rapid and accurate identification of FCSO structures.

Recent developments in solid-state NMR (ssNMR) and dynamic nuclear polarization (DNP) have enabled studies of plant cell wall architecture even without ^13^C labelling (Wang et al., 2016; Zhao et al., 2021; Kirui et al., 2022; Cresswell et al., 2025). Similar approaches can be applied to brown algal cell walls to analyse their native polysaccharide composition and organization without extraction, allowing structural investigation of complex matrices rich in alginate, cellulose, and fucoidan. Using multidimensional (2D—3D) correlation experiments, ssNMR can identify intermolecular interactions and spatial proximity between polysaccharides, analogous to studies in plant cell walls (Wang et al., 2016). It also provides insight into polymer conformation, crystallinity, and dynamics, highlighting the potential structural role of fucoidan in the cell wall compared to its secreted form. Additionally, ssNMR can probe interactions between polysaccharides and other components, such as polyphenols, which have been observed to be covalently bound to extracted FCSPs (Birgersson et al., 2026), similar to lignin–carbohydrate interactions in terrestrial biomass (Kang et al., 2019; Cresswell et al., 2025). Together, these approaches make ssNMR a powerful tool for elucidating the structural role of fucoidan in brown algal cell wall.

Mass spectrometry (MS) is an extremely sensitive technique that provides high-resolution in structural information from minute sample quantities. MS-based approaches have been considered for the characterization of fucoidans, in particular MALDI-TOF (Matrix-Assisted Laser Desorption/Ionization Time-of-Flight), AP-MALDI-Orbitrap (Atmospheric Pressure Matrix-Assisted Laser Desorption/Ionization source with an Orbitrap mass analyzer), and ESI-MS (Electrospray Ionization Mass Spectrometry) to get insights into monosaccharide composition, sequence of monosaccharides, linkage groups and sulfation patterns (Anastyuk et al., 2015; Zayed et al., 2020; Sichert et al., 2021). The upper detection limit for oligosaccharides in MALDI-MS is approximately 2 kDa, where the fragment can still be annotated confidently (Reyes-Weiss et al., 2024). However, glycans in general do not efficiently ionize. This issue has been addressed for sulfated fucans by coupling MS with separation techniques such as ion-pair reversed-phase liquid chromatography (IP–RPLC) (Nikolić-Chenais et al., 2022; Ropartz et al., 2022), which also allowed to investigate FCSP structures of low abundance. Limitations in the use of MS approaches to analyse fucoidans occur, including those associated with the labile nature of the sulfate group. This can result in the desulfation of monosaccharide sub-units during the preparation of the samples for MS analysis. However, advanced fragmentation techniques have recently been developed, including for sulfated glycans. For FCSPs, tandem MS (MS-MS) has been applied, with the highly energetic helium charge transfer dissociation (He-CTD) technique, which efficiently fragment complex oligosaccharides, generating cross-ring fragments while preserving labile modifications such as sulfation (Ropartz et al., 2022). The highly energetic fragmentation methods outperformed traditional collision-based approaches for sulfated glycans (Ropartz et al., 2015; Klein et al., 2019). More recently, the package GlycoAnnotateR has been developed for de novo annotation of glycan composition based on MS data, including for fucoidans (Bligh et al., 2025).

As a result, NMR and MS are highly complementary, and combining the two approaches is likely to improve the overall quality of a study and to enhance the coverage of FCSP's diversity. Generating an empirical reference data based on both MS and NMR would benefit the broader community, by enhancing the characterization of unknown FCSP/FCSO structures and linking them to their biological activities. However, both analytical approaches, typically require an initial depolymerization step. In some cases, NMR can still identify recurring structural motifs within FCSPs (Birgersson et al., 2024). The use of enzymatic hydrolysis is ideal for FCSPs, to avoid alteration of the sulfation pattern. Yet, while the number of characterized enzymes degrading FCSPs increases, most are targeting sulfated fucans and their number is not yet matching the complex diversity of the structures to be identified.

**Fig. 3:**
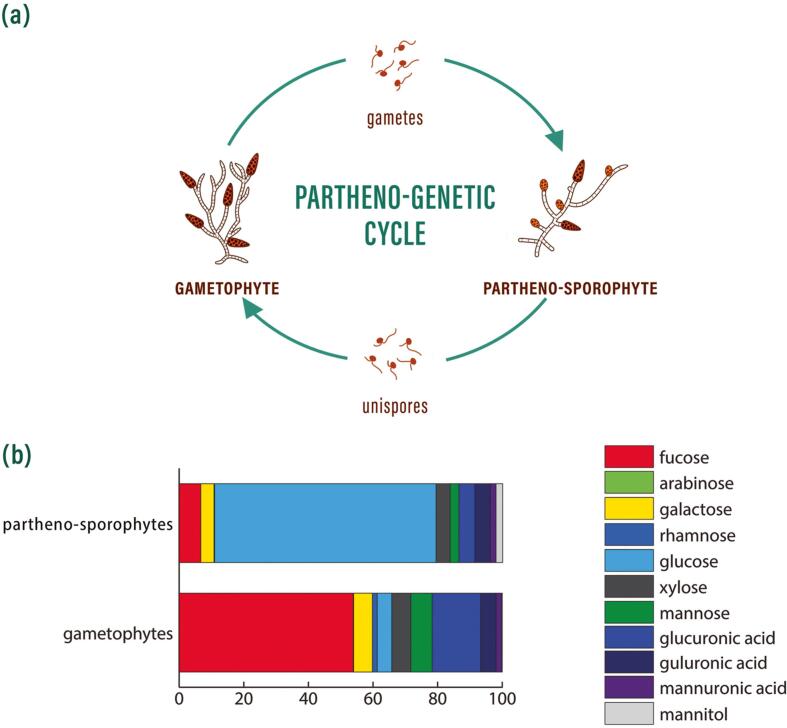
Snapshot of fucoidan composition at different life stages of the model brown alga *Ectocarpus* species. 7. **A,** Life cycle of *Ectocarpus* species 7. **B,** Relative composition of monosaccharides (% of the extract) in hot water extracts of fucoidans from two Ectocarpus life stages determined by acid hydrolysis and high-performance anion-exchange chromatography with pulsed amperometric detection (Birgersson et al., 2024). All samples were run in triplicates. (For interpretation of the references to colour in this figure legend, the reader is referred to the web version of this article.)

*Ectocarpus* species 7 has been developed as a genetic and genomic model to study the biology and evolution of brown algae (Cock et al., 2010; Coelho, 2024). In this context, this alga has been extensively investigated during the last 20 years, and its life cycle is well understood and controlled under laboratory conditions (Fig. 3A). A major advance was made recently with the implementation of the CRISPR-Cas9-based mutagenesis technology in *Ectocarpus* cells (Badis et al., 2021). Although FCSPs have not been investigated yet in this species, some information is available from the closely related *Cladosiphon okamuranus* (Nagoaka et al., 1999; Sakai et al., 2003) and *Chordaria flagelliformis* (Bilan, 2008). However, it is not known how conserved or variable the structure and composition of FCSPS may be across these Ectocarpales species. This knowledge gap is currently a limiting factor in understanding FCSP biosynthesis and functions if a genetic approach was to be considered. Brown algal species of economic importance have been better explored regarding their FCSP content, with for instance *Saccharina japonica* (Jin et al., 2020), *Laminaria hyperborea* (Kopplin et al., 2018), *Saccharina latissima* (Tran et al., 2022; Birgersson et al., 2024) or *Alaria esculenta* (Birgersson et al., 2024). For these species, only the large and commercially exploited sporophytic phase was analysed, not the microscopic gametophytic phase. While this is currently of no impact for the seaweed industry, it might limit its future expansion. Indeed, while sporophytes are commercially exploited, the hatcheries currently established in Europe are based on biobanking gametophytes, and little is known about the heritability of key traits, including the yield and composition of exploited polysaccharides. Our initial investigation of the two main stages of the life cycle of *Ectocarpus* species 7 indicates that FCSP composition might differ strongly across its different phases (Fig. 3B). How this relates to distinct structures and/or relative abundance is under investigation in our laboratories. If such differences can be observed in life stages being nearly isomorphic such as in Ectocarpales, they surely can also be expected in species with stronger dimorphisms during life cycle progression such as Laminariales (Fig. 2).

In addition to variations across different stages of the life-cycle progression, factors such as taxonomy, location, developmental stages, and abiotic stresses have been shown to impact FCSPs composition and structures (Fig. 2) (Deniaud-Bouët et al., 2017; Kloareg et al., 2021). However, such variations have usually been reported at the level of the whole algal plant, on fractionated walls, with the subsequent loss of most spatial and developmental information. Therefore, the fine-tuning of FCSP structures and macromolecular configurations in developing tissues is largely unknown. The use of metachromatic stains on tissues is not specific enough to offer a conclusive view of glycan distribution (Mariani et al., 1985; Bisgrove and Krop 2001; Mazéas et al., 2023). Anti-FCSP monoclonal antibodies (mAbs) are appropriate for mapping epitopes in tissues and to establish their structure/function relationships. There are currently four specific mAbs targeting FCSPs from Fucales available at the SeaProbes service, that hosts and maintains cell lines producing Brown Algal Monoclonal (BAM) antibodies (Torode et al., 2015 and 2016). No well-characterized antibodies have been generated against FCSOs of the major brown algal model Ectocarpales and Laminariales species yet.

A complementary approach to the production of novel antibodies would be the implementation of metabolic click labelling for the glyco-imaging of intact cells in brown algae. Indeed, click chemistry has proven useful in tracking the metabolic incorporation of modified sugars in polysaccharides in living organisms (Voiniciuc et al., 2018; Anderson et al., 2012; Daughtry et al., 2020). For this approach, peracetylated reporters are added in the surrounding media. After crossing the plasma membrane and being deacetylated intracellularly, these reporters are incorporated into polysaccharides. The labelled biopolymers can then be visualised through click addition to a fluorescent probe based on azide-alkyne cycloaddition. So far, this methodology has rarely been used in aquatic species but was successfully applied recently to green and red seaweeds (Delpont et al., 2025). However, not all monosaccharide reporters can be efficiently incorporated (Zhu and Chen, 2017), and the complex cellular metabolic machinery may convert the functionalized monosaccharide into other products, resulting in labelling outside the targeted biopolymers (DeVree et al., 2021). Interestingly, new metabolic labelling-based approaches are emerging, including carbohydrate hydrogel-probes that may bind alginates in brown algae (Besten et al., 2025), providing relevant future avenues for exploring the structural diversity of FCSPs.

## 3 The production of FCSOs inspired by nature

**Fig. 4:**
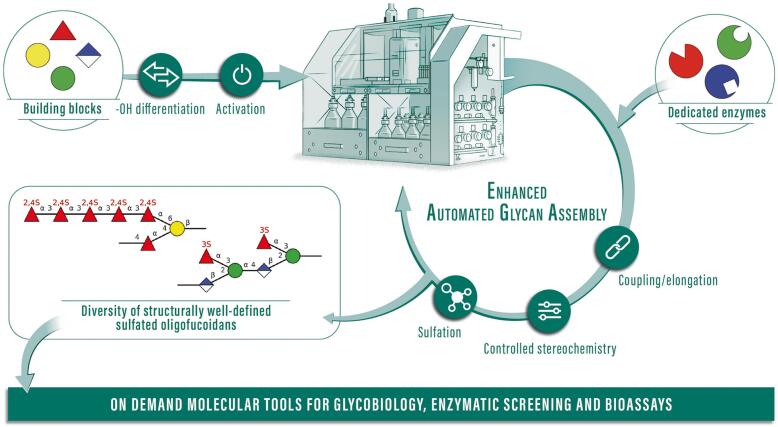
Description of Automated Glycan Assembly (AGA) to support different streams of glycobiology research. Simple monosaccharidic building blocks functionalized via hydroxyl differentiation serve as glycosyl donors and acceptors. Upon activation, these intermediates undergo stereo-controlled chain elongation, possibly assisted by biocatalysts. Automation of coupling, sulfation, and deprotection steps enables rapid access to diverse, well-defined oligo- and polysaccharides, supporting both fundamental research and high-value applications in glycoscience.

The demand for structurally well-defined oligosaccharides in the context of high-value applications is growing significantly. Access to such FCSOs would advance both research and practical applications of FCSPs (Fig. 4): i) they will serve as standards for the structural characterization of unknown glycans and for the screening of mAbs epitopes [79]; ii) they will enable the discovery and exploration of substrate preference of enzymes involved in FCSPs biosynthesis (glycotransferases, GTs) and re-modelling (sulfotransferases, STs; sulfatases, SAs); iii) they will support the biocatalytic synthesis of FCSOs for bioactivity testing. One approach to obtain structurally well-defined FCSOs, in particular for research purpose, is through the production of synthetic oligosaccharides, which is typically addressed by chemical synthesis. In general carbohydrates chain elongation requires advanced and time-consuming protection/activation/deprotection strategies due to subtle differences in reactivity from substrates with otherwise close reactivities. Therefore, couplings of carbohydrates have to be tailored according to each targeted final product to ensure both regio- and diastereoselectivities (Fig. 4).

Efficient approaches based on automated glycan assembly (AGA) have recently been described for the synthetic production of FCSOs up to 20-mer (Crawford et al., 2024; Chen et al., 2025; Mardhekar et al., 2025). In contrast with automated peptide synthesis, there is currently limited availability of reagents with controlled reactivities to further develop AGA of oligosaccharides, so that the design and implementation of schemes for glycosylation and subsequent molecular modulation, including sulfation, is still limited to specialists. However, the systematic prediction of the reactivity of required molecular partners for AGA has significantly contributed to the development of this approach. For instance, the acceptors can be defined with relative reactivity values descriptors (Cheng et al., 2018), and prediction of the temperature range is useful to activate acceptors under optimal conditions (Lin et al., 2024). Moreover, the use of pre-activation-based multicomponent one-pot glycosylation has allowed the synthesis of complex glycans up to 1080-mer (Yao et al., 2022). Bio-inspired activation and the use of organometallic catalysts have also contributed to enriching the current chemical toolbox. This allows a better tuning between donors and acceptors, limiting the manipulation of protecting groups, and increasing regio- and diastereoselectivities (McClary and Taylor, 2013; Taylor, 2015; Rao et al., 2023; Warnes and Fascione, 2024; Hao et al., 2025; Sebastian and Loh, 2025). Recently, it was shown how sulfate groups, which are usually introduced on the glycosides at the end of the process, helped in the orientation of the glycosidic coupling, thus strengthening the wide range of resources available to chemists (Maki et al., 2025).

Alongside strict chemical approaches, chemoenzymatic synthesis of complex carbohydrates has also been developed (Liao et al., 2023; Lin et al., 2024). The recognition and predefined orientation of the donor and acceptor in the biocatalytic pocket ensure both the activation of the two reactive entities and very high selectivity. This results in drastic reduction of the number of protection and deprotection steps, and a more sustainable process. Two families of enzymes allow specific preparation of fucosylated glycosides. The fucosyltransferases have the advantage of being highly selective (Li et al., 2019; Tsai et al., 2019; García-García et al., 2020; Zhong et al., 2021; Ali et al., 2023; Khaled et al., 2025), but require the expensive GDP-fucose as fucosyl donor. This drawback can be overcome by using multi-enzymatic systems (del Amo 2010; Zhong et al., 2021). For instance, the fucosylation of the sialyl Lewis X tetrasaccharide required six biocatalysts for phosphorylation of fucose, synthesis and transfer of the GDP-fucose donor, recycling of the released ATP and GDP, and removal of the inhibiting inorganic diphosphate. The fucosynthases are hydrolytic enzymes mutated in such a way that they have lost their native hydrolytic activity (fucosidase) but have preserved their capacity to transfer fucosyl residues when a suitable synthetic donor is provided. The main donors are generally simple monofucosides, i.e. the *p*-nitrophenyl or fluoride derivatives (Sakamura et al., 2012; Herman et al., 2008). Many fucosyl-containing oligosaccharides have been synthesized for human health purposes using such fucosynthases (Sakurama et al., 2012; Del Amo et al., 2010; Li et al., 2017; Tsai et al., 2019). Once produced, the polyfucosidic chain is decorated with sulfate groups.

It is worth mentioning that current yields obtained by chemical or chemo-enzymatic approaches are low, in the ten-mg scale, which can be relevant for laboratory studies but is not suitable for large scale analysis and industrial applications. Several directions should be explored to improve and increase the production of well-defined sulfated oligofucoidans by chemo-enzymatic approaches (Fig. 4). Firstly, the synthesis of the fucan backbones requires more efficient and scaled-up chemical and enzymatic methods. Secondly, there is a need to develop 3′-phosphoadenosine-5′-phosphosulfate (PAPS) chimera and/or to design novel related catalytic systems. Indeed, both existing substrates and multi-enzymatic systems are expensive and unsuitable for high-throughput experiments (Wang et al., 2023). New molecular and enzymatic designs are required for studying the regioselectivity of sulfotransferases and for efficient chemoenzymatic synthetic approaches on a larger scale. Thirdly, the characterization of brown algal STs involved in the re-modelling of FCSPs will provide biocatalysts to be used for regiospecific sulfation of various fucan backbones. Finally, the use of solid-supported technologies would simplify purification steps to obtain structurally well-defined FCSOs.

## 4 Unlocking the fucoidan biosynthetic and re-modelling pathways

Unlike genomes, transcriptomes, or proteomes, glycomes are produced in a non-templated manner, relying on spatially and temporally well-organized multiple enzyme activities. In contrast to GAGs, we currently have limited understanding of the molecular bases of the biosynthesis and re-modelling of FCSPs, as no GTs, STs, and SAs involved in these metabolic processes have been fully biochemically characterized yet.

The publication of the first genome of a brown alga, *Ectocarpus* species 7 in 2010 (Cock et al., 2010) laid the ground to identify candidate genes coding for these three types of enzymes (Michel et al., 2010). Candidate genes for GTs, STs, and SAs potentially associated with FCSPs biosynthesis and remodelling were found to belong to multigenic families. The multiplicity of these genes may reflect the need for assembling different monosaccharide units, and their sulfation at different positions. Since then, the study of brown algae has entered a new era as considerable effort have contributed to establish new and substantial genetic and genomic resources for these organisms. The large genomic resource generated through the recent Phaeoexplorer project (https://phaeoexplorer.sb-roscoff.fr^;^ Denoeud et al., 2024) has provided insights on the evolutionary history of the lineage, including on their polysaccharidic metabolic pathways. These new genomes have enabled expanding the prediction of GTs, STs, and SAs to other brown algae, in particular kelps (Mazéas et al., 2024). GTs are distributed among 37 families in brown algae, with low gene redundancy, with an average of 3 genes identified per family. Some families are larger, such as the GT2 (16 genes on average), GT4 (16), GT31 (9), and GT47 (9). However, the family size does not predict their involvement in the synthesis of a given polysaccharide. More advanced predictions have been made specifically for fucan, with fucosyltransferase candidate genes likely being restricted to families GT10, 23, 41, and 74 (Mazéas et al., 2024). None of these GT members have been biochemically characterized yet, so their possible involvement in FCSPs synthesis is still highly speculative. Similarly, 10 major orthologous groups of STs have been identified in brown algae but not all would be expected to be active on carbohydrate substrates. The SAs shape a smaller family, with 7 genes on average per genome, all belonging to the S1_2 family. As for the GTs, no functional genomics evidence has been published yet for STs and SAs. Indeed, two major challenges have hinder studying metabolic pathways in brown algae so far: i) the lack of genetic method to mutate genes and impair metabolic function(s); ii) the low efficiency of heterologous expression of brown algal to support the purification of recombinant proteins for biochemical and structural analysis.

Importantly, CRISPR-Cas9-based approaches have now been implemented with success in *Ectocarpus* species 7 (Badis et al., 2021), in *Saccharina japonica* (Shen et al., 2023) and very recently in *Scytosiphon promiscuous*, *Laminaria digitata* and *Undaria pinnatifida* (Martinho et al., 2025). However, these approaches still have to be used in the context of deciphering metabolic pathways. As mentioned above, GTs, STs and SAs are part of multigenic families, making it difficult to predict which gene codes for enzymes involved in specific transfer of monosaccharide units and in site-specific sulfation and/or desulfation. Functional redundancy among these enzymes should not be ruled out too. In this context, the outcomes of genetic analysis based on the CRISPR-Cas9 approach is currently unpredictable and should point towards determining specificity of targeted enzymes before engaging into it.

Another challenge is the expression of brown algal genes in heterologous systems. So far, approximately 50 genes of brown algae have been expressed in heterologous hosts, and most of them are from *Ectocarpus* species 7 and *S. japonica*, potentially because both species were the first for which high quality genomes were obtained. The main heterologous expression system used so far has been the bacterium *Escherichia coli* for subsequent protein purification, and other systems have been considered, including microbial (*Cupriavidus necator*, *Saccharomyces cerevisiae*, *Pichia pastoris*) and multicellular (*Arabidopsis thaliana*, insect cells) hosts for functional studies. Interestingly, the first purification of recombinant GT and ST from *Ectocarpus* species 7 from *E. coli* has been recently reported (Zayed et al., 2025). However, the yield of recombinant enzymes was very limited, and no activity assays on specific substrates were performed. Human Embryonic Kidney 293F (HEK293F) cells have been shown to be particularly suitable for expressing eukaryotic GTs and represent an interesting platform for the characterization of brown algal GTs (Prabhakar et al., 2020). However, any additional hosts and relevant technology, including modelling prediction of targeted enzymes before cloning, may be useful to unlock the heterologous expression of brown algal proteins.

Biochemical characterization of recombinant proteins, in particular GTs and STs, will involve testing their specificity against natural and synthetic oligosaccharides using commercial kits. Products of reactions will be further analysed, including by NMR and/or MS techniques as mentioned in sections above, to determine the structure of the products. In addition, glycoarray using well-defined synthetic oligos and/or pure natural FCSOs would help to improve the throughput for screening GTs and STs enzymes. The use of these arrays should be combined with chemically functionalised sugar-nucleotide donors to enable direct detection of the acceptor after transfer of the modified glycosyl residue (Ruprecht et al., 2020). While it is unclear if such non-natural donors can be efficiently accepted by the enzymes, small modifications including alkynyl- and azido-modifications are usually well tolerated by GTs as previously observed across metabolic glycan labelling studies in bacteria (Dumont et al., 2012), mammals (Briard et al., 2018) and plants (Ruprecht et al., 2020).

## 5 Advancing knowledge on fucoidans for the benefit of society

A better understanding of fucoidan metabolism and the development of analytical tools for these polysaccharides would have positive consequences on different streams of research, including to better understand their structure-function-bioactivity relationships in the context of medical and pharmaceutical applications to develop regulated therapeutics, to assess their importance in the marine carbon cycle, and to develop seaweed biotechnology (Fig. 2).

### 5.1 Medical applications - structure-bioactivity-function relationships and quality of FCSPs/FCSOs

Stemming from their ability to mimic sulfated GAGs, FCSPs/FCSOs exhibit a wide array of bioactivities, modulated by their monosaccharide composition, molecular mass and sulfation patterns. Numerous reviews offer a comprehensive view to the biological properties of FCSPs/FCSOs and the reader is directed to these resources for dedicated information (Deniaud et al., 2017; Torres et al., 2020; Zayed and Ulber, 2020; George and Shrivastav, 2023; Zheng et al., 2025). For example, it was observed that FCSPs/FCSOs anti-inflammatory and antioxidant activity increased with sulfate content of low molecular mass fucoidans, while anti-lipogenesis decreased (Chen et al., 2021). For brown algae, establishment of structure−function relationships would be possible only if pure and defined FCSOs are used. Attempts to obtain sufficient quantities and consistent natural FCSOs would require scaling-up extractions and developing/adapting appropriate environmentally friendly reproducible protocols (Chadwick et al., 2025). An alternative would be the use of tailormade FCSOs obtained through chemical or chemoenzymatic approaches as described above, and this should be prioritized to advance understanding on structure/function relationships.

The assessment of the anticoagulant activities of 58 synthetic oligofucans, bearing either α-1,3-linkages or alternating α-1,3- and α-1,4-linkages, and with various patterns of sulfation, was recently described (Chen et al., 2025). It showed that only a selection of structures selectively inhibited the intrinsic coagulation pathway, with the 2,3,4-O-tri-sulfated oligosaccharides having strong anticoagulant effects. In a different study, the chemical synthesis of low molecular weights fucoidan analogue and the testing of their activities on heparanase and SARS infection showed that fucoidan derivatives without sulfate groups have no inhibitory activity against heparanase and low affinity to SARS-CoV-2-S protein (Sugimoto et al., 2025). In sharp contrast, fucoidan derivatives in which all hydroxyl groups were sulfated showed high heparanase inhibitory activities and a high affinity for SARS-CoV-2-S. A different approach has been explored previously to make easier access to synthetic sulfated oligofucosides and mimic natural polysaccharides, relying on the production of glycopolymers multi-presented sulfated fucosyl units with well-defined sulfation patterns (Fan et al., 2018). These synthetic mimetics showed inhibitory activities against the multiplication of the influenza A virus, which were related to their sulfation patterns rather than their sulfation degree. As such, the mimetics bearing a 2-OH sulfation on their fucosyl units were inhibiting both the H1N1 and H3N2 viruses, whereas those bearing a 3-OH sulfation were only inhibiting the H3N2 virus.

New insights into the structure and function of FCSPs/FCSOs could be applied in advancing therapeutic development and glycomedicine. As an example, progress in cancer therapy has been slow because of the rare in vivo investigations using pure fucoidans of known structures. Most studies have been performed on rodents, using fucoidans through injections or as food supplement. Positive impacts have been observed on lung cancer (Lee et al., 2012; Hsu et al., 2017), colon cancer (Han et al., 2015); leukaemia (Haneji et al., 2005; Maruyama et al., 2006), and angiogenesis (Liu et al., 2012; Chen et al., 2015). Besides those studies, clinical trials are rare. A search conducted on https://clinicaltrials.gov/ (latest access on 19.11.2025) with the term “fucoidan” reported 23 studies with different status. Most of those involved cohorts too small to give significant outputs (〈100). Overall, these studies suggest that extracted FCSPs, tested as dietary supplement with other chemotherapeutic drugs, or even as synergetic antitumor drugs, can enhance the action of co-treated drugs, reduce patient fatigue or improve patient prognosis. If FCSPs had to be provided through direct consumption of brown algae, adverse effects such as liver toxicity, diarrhoea, iodine exposure, driven by other algal components, should not be ignored. In most of those trials, extracted fucoidans were provided as part of the diet in quantities of around 4–6 g per day for a few weeks. If assumed that fucoidans make ∼9–14% of the dried algal biomass and that dried seaweed represents 20% of fresh biomass, this would represent the consumption of ∼150–300 g of fresh seaweed per day.

An additional parameter worth investigating is the fate of FCSPS in a cellular and tissue context. Indeed, while transducing signals seem to be induced through the recognition of active FCSOs by cell surfaces, their incorporation by the cell, their fate at a subcellular level and their lifetime, are unknown. Getting such knowledge would help to gain a better understanding of FCSO bioactivities, but will require the development of dedicated tools. Labelled FCSOs would be particularly useful for their tracking. However, the use of the aforementioned anti-FCSP mAbs could also help investigations at greater scales, as illustrated by the penetration abilities of fucoidans from various sources inside osteosarcoma cells (Gupta et al., 2020).

### 5.2 Seaweed farming – FCSPs biosynthesis and characterization as part of kelp breeding programs

Advancing knowledge on the FCSP metabolic pathway through the characterization of GTs, STs, and SAs could contribute to gain a better understanding of kelp cultivars to be considered for FCSPs and FCSOs production as the genes coding for these enzymes could be used as markers for molecular breeding. The cultivation of kelp species, and especially of *S. latissima*, consists of a nursery stage on land for the development of microscopic gametophytes, and the subsequent development of juvenile sporophytes for seeding on substrates deployed at sea (Saether et al., 2024). Tools to support molecular breeding are developing, including collection and preservation of gametophyte stock resources, establishment of mapping populations, construction of genetic linkage maps, and development of DNA markers in both model and economically important brown algae (Shan and Pang, 2021; Nehr et al., 2025). In this context, it is important to keep in mind potential biochemical redundancy among these genes/enzymes. In addition, specific anti-fucoidan antibodies could be used for phenotyping parent strains and their progeny. Such markers and tools could also be used to investigate trait inheritability between gametophyte and sporophyte. Additional parameters to be measured as part of breeding program(s) would include monosaccharides composition and sulfate level in extracted fucoidans. This would require setting-up a reliable and reproducible method of extraction as it has been shown that fucoidan extraction and fractionation methods influence the quantity and composition of the fucoidan fraction (Birgersson et al., 2025). Another convenient (quick, cheap, and non-invasive) option would be the use of Fourier Transform Infrared spectroscopy (FT-IR) and Raman spectroscopy for phenotyping. However, no reference database has been created yet to infer the absorption bands of specific components for brown algae, although some attempts have been made previously to analyse brown algal biomass by FT-IR (Gómez-Ordóñez and Rupérez, 2011; Beratto-Ramos et al., 2020; Li et al., 2023; Fidai et al., 2024).

### 5.3 Environment – Importance of FCSPs in the marine carbon cycle

Fucoidans have been suggested to contribute to carbon removal and sequestration by brown algae (Buck-Wiese et al., 2023; Hellige et al., 2026). Quantification of such a contribution was based on monosaccharide quantification, antibody binding, anion exchange chromatography, and enzymatic hydrolysis to detect and quantify fucoidans. Expanding the range of antibodies directed against fucoidans would provide useful tools to follow the fate of FCSPs in the environment. More generally, estimates of seaweed-related carbon capture and sequestration vary widely (61–268 Tg of carbon per year) (Krause-Jensen and Duarte, 2016). Getting a more detailed knowledge of the FCSP-contribution in this carbon pool would help to better define some of the estimates.

## 6 Concluding remarks and recommendations

Algae are essentially photosynthetic eukaryotes that occupy multiple branches of the tree of life and are vital for planet function and health. Brown algae provide core ecosystem services integral to human activities. Fucoidan are complex molecules, and their study has been hindered by several biological and technical challenges. Therefore, it is key to consider recent technical progress, newly available in silico resources and biological tools to go beyond our current stage of knowledge and advance Glycoscience. New annotated genomes of brown algae are available, providing relevant genes for functional characterization of enzymes potentially involved in synthesis and remodelling of fucoidans. Transformation has long been a bottleneck in brown algae, but CRISPR-Cas9-based technology is now available in several brown algal species to support in vivo characterization of candidate genes. These biological tools can be combined and leveraged with new chemical tools. These include NMR, MS, and analysis of glycosidic linkages for determining the composition and structure of polysaccharides; automated synthesis of oligosaccharides to produce standard for FCSPs structure elucidation and substrates for characterization of enzymes involved in fucoidan metabolism; immunolabelling and metabolic click labelling for visualisation of polysaccharides in cell biology experiments and for the detection of products of enzymatic reactions. In addition, recent advancements in AI can support analysis of data in the context of fucoidan research, in particular to go deeper in elucidating their structure in a wider diversity of brown algae. Despite numerous reports on their bioactivities, FCSPs/FCSOs have not been developed as regulated therapeutics yet, as major bottlenecks remain regarding the consistent access to pure active forms and their comprehensive chemical characterization. Similarly, biochemical characterization of enzymes will require well-defined substrates, and methods for identifying the reaction products.

**Fig. 5:**
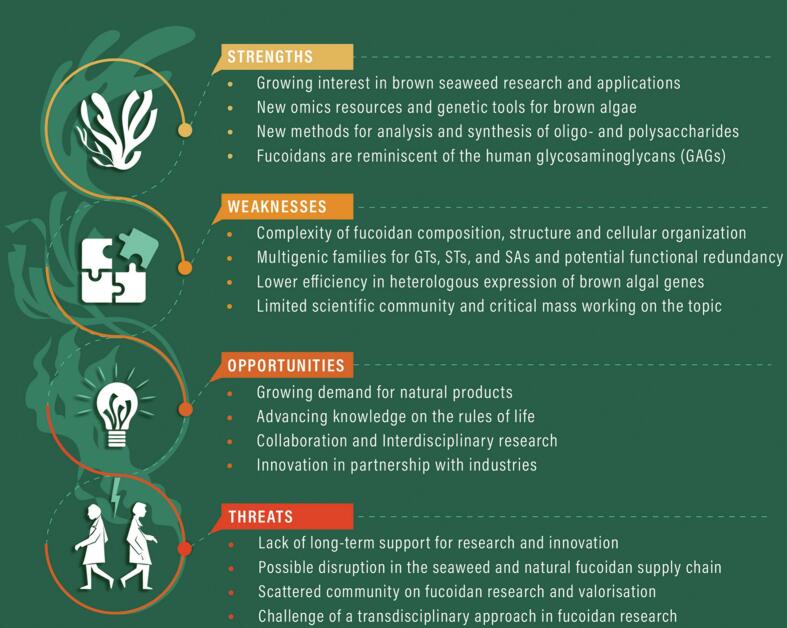
Strengths, Weaknesses, Opportunities, and Threat (SWOT) analysis associated with current and future fucoidan research.

Interdisciplinarity and synergy would be key as tackling these problems would require strong and complementary expertise in biology, biochemistry, enzymology, carbohydrate and protein structural elucidation, and synthetic chemistry. Our SWOT (Strengths, Weaknesses, Opportunities, Threats) analysis identified great innovation potential despite current limitations linked to fucoidan's complexity and a scattered research effort (Fig. 5). Advancing our understanding of fucoidans and of their functional implications is pivotal for unlocking their full industrial potential. By integrating this knowledge into seaweed farming strategies, species could be farmed with optimized fucoidan profiles, ensuring consistent quality and targeted bioactivity. This alignment between sustainable cultivation and industrial utilization would not only supports the growing demand for high-value products in pharmaceuticals, nutraceuticals, and cosmetics but would also promotes environmentally responsible resource management.

## CRediT authorship contribution statement

**Thierry Tonon:** Writing – review & editing, Writing – original draft, Project administration, Conceptualization. **Finn L. Aachmann:** Writing – review & editing, Writing – original draft, Conceptualization. **Vincent Ferrières:** Writing – review & editing, Writing – original draft, Conceptualization. **Cécile Hervé:** Writing – review & editing, Writing – original draft, Visualization, Funding acquisition, Conceptualization.

## Declaration of competing interest

The authors declare that they have no known competing financial interests or personal relationships that could have appeared to influence the work reported in this paper.

## Data Availability

No data was used for the research described in the article.
